# Type-II Fuzzy Decision Support System for Fertilizer

**DOI:** 10.1155/2014/695815

**Published:** 2014-04-30

**Authors:** Ather Ashraf, Muhammad Akram, Mansoor Sarwar

**Affiliations:** ^1^Punjab University College of Information Technology, University of the Punjab, Old Campus, Lahore 54000, Pakistan; ^2^Department of Mathematics, University of the Punjab, New Campus, Lahore 54590, Pakistan

## Abstract

Type-II fuzzy sets are used to convey the uncertainties in the membership function of type-I fuzzy sets. Linguistic information in expert rules does not give any information about the geometry of the membership functions. These membership functions are mostly constructed through numerical data or range of classes. But there exists an uncertainty about the shape of the membership, that is, whether to go for a triangle membership function or a trapezoidal membership function. In this paper we use a type-II fuzzy set to overcome this uncertainty, and develop a fuzzy decision support system of fertilizers based on a type-II fuzzy set. This type-II fuzzy system takes cropping time and soil nutrients in the form of spatial surfaces as input, fuzzifies it using a type-II fuzzy membership function, and implies fuzzy rules on it in the fuzzy inference engine. The output of the fuzzy inference engine, which is in the form of interval value type-II fuzzy sets, reduced to an interval type-I fuzzy set, defuzzifies it to a crisp value and generates a spatial surface of fertilizers. This spatial surface shows the spatial trend of the required amount of fertilizer needed to cultivate a specific crop. The complexity of our algorithm is *O*(*mnr*), where *m* is the height of the raster, *n* is the width of the raster, and *r* is the number of expert rules.

## 1. Introduction

Combining fuzzy sets and logic with geospatial information opens up a new dimension for scientist. Natural phenomena are mostly fuzzy in nature. They show a degree of uncertainty or vagueness in their extent and attribute, which cannot be expressed by a crisp value. Fuzzy sets, introduced by Zadeh [1], provide a mechanism for communication between computing systems and humans [2]. A fuzzy control system is developed on the basis of fuzzy set theory and fuzzy logic [3]. Many fuzzy inference systems and defuzzification techniques have already been developed. These techniques are useful in obtaining crisp output from a fuzzy input. The crisp output values are calculated using fuzzy rules applied in an inference engine using defuzzification methods [4].

A fuzzy logic system (FLS) includes a fuzzifier, an inference engine, and a defuzzifier [5]. Quite often, the knowledge used to construct the membership function is uncertain. These uncertainties can occur due to the following [6]:

- the word used in defining the membership function of antecedent and consequent of rules means different to different people;
- the training data set may be noisy;
- the classification used to define these words can be expressed by different people as different functions, that is, trapezoidal, triangle, or Gaussian.

All these uncertainties should be translated into the membership function of antecedent and/or consequent. Type-I fuzzy sets cannot model these uncertainties [7]. This is due to the crisp nature of their membership function. In 1975, Zadeh introduced the concept of type-II fuzzy sets [8]. Type-II fuzzy sets are an extension of type-I fuzzy sets in which membership functions are themselves type-I fuzzy sets. They are very useful in those scenarios where it is difficult to find a crisp or exact membership function.

Type-II fuzzy sets provide an additional degree of freedom in Mamdani and Assilian [9] and Takagi-Sugeno-Kang (TS) [10] fuzzy inference systems. These inference systems can be very useful where a lot of uncertainties are present [11]. Fuzzy type-II sets have the potential to provide more flexibility to tackle uncertainties and hence give better results and performance, for example, [12–15].

Gale [2], Pipkin [16], and Leung [17] were pioneers in introducing the fuzzy logic in the geographical domain. Much work has been published on the use of fuzzy logic in a spatial domain especially in land suitability modeling [18–23]. Most of them model a membership function through (1) a deterministic formula, (2) the use of a regression equation, or (3) the use of clustering procedure like fuzzy c-mean. Fertilizer, one of the important elements in crop yield, is an organic, inorganic natural, or synthetic material, when added to a soil surface, it provides one or more nutrients which are essential for the plants growth [24]. It is estimated that around 30%–50% of crop production are attributed to fertilizer [25]. The use of fertilizer depends on the soil nutrient spatial variation. Soil spatial variation is expressed as membership in soil classes [26]. Generally, membership in soil classification is derived in two ways [27], that is, classification derived from data and knowledge based semantic model. Lagacherie [28] proposed a method to translate soil class description which is based on possibility theory and fuzzy pattern matching. Qi et al. [29] developed a prototype based on soil knowledge as fuzzy membership functions which is used in fuzzy soil mapping system. Zhu et al. [19] present a method using descriptive knowledge to construct fuzzy membership functions on soil-landscape relationships. Membership function was derived from 22 field samples collected using purposive sampling approach. Zhu et al. [20] predict soil properties using fuzzy membership values. Their results show that regression with fuzzy membership values can be used where soil terrain relationships are more complicated. Bogataj et al. [21] also used regression analysis with fuzzy approach to land valuation. They effectively use a fuzzy membership function for determining the independent variable of the regression function.

In this paper we have introduced a fuzzy decision support system for modeling a spatial surface of fertilizer. This work is an extension of fuzzy system based on type-I fuzzy sets. There was an ambiguity in defining the membership function of nutrients in type-I fuzzy sets. To overcome this ambiguity, we developed the membership function of nutrients based on type-II fuzzy sets.

The paper is divided into four sections. The first section describes the preliminaries of type-II fuzzy set. The second section describes the basic structure and algorithm of our system. The third section explains the different parts of our proposed fuzzy system, that is, fuzzifier, fuzzy inference engine, and defuzzifier. The fuzzifier uses a membership function and the foot print of uncertainty based on the classification of the nutrients defined by Soil Fertilizer Research Institute (SFRI) of Pakistan. SFRI has a number of laboratories all over the country, which takes soil sample from farmers, test the nutrient composition of the sample, and suggest fertilizers based on these 95 rules that have defined in our fuzzy inference engine. The last section is on conclusions and future research direction.

## 2. Preliminaries

### 2.1. Type-II Fuzzy Set

Type-II fuzzy sets generalize type-I fuzzy sets to handle more uncertainties. This is done by using fuzzy membership functions. Zadeh [8] defines type-II fuzzy sets that have membership functions that themselves are fuzzy set. He explains this change as:* “motivated by the close association which exists between the concept of a linguistic truth with truth-values such as true, quite true, very true, more or less true, and so forth, on the one hand, and fuzzy sets in which the grades of membership are specified in linguistic terms such as low, medium, high, very low, not low and not high, and so forth, on the other.”*

**Definition 1 (fuzzy sets)**

Adopted from [1], a fuzzy set, *A*, defined over universe *X*, is a function defined as follows:

(1) $$\begin{array}{c} A:X⟶U,\\ x⟼u_{x}, \end{array}$$

where *x* ∈ *X*, *U* = [0,1] is a unit interval and *u*_*x*_ ∈ *U* is the membership grade of the element *x* in the fuzzy set *A*.

**Definition 2 (type-II fuzzy sets)**

Adopted from [30], a type-II fuzzy set, $\tilde{A}$, over the universe *X*, is defined by the following function:

(2) $$\begin{array}{c} \tilde{A}:X⟶F\left(U\right), \end{array}$$

where *U* = [0,1] is the domain of membership.

There are two categories of type-II fuzzy sets (1) generalized and (2) interval type-II fuzzy sets. The difference between the two categories is in the way of modeling the fuzzy membership function. The former models the fuzzy membership grade as a fuzzy number between zero and one, whereas the later models the fuzzy membership grade as a crisp interval between zero and one.

**Definition 3 (generalized type-II fuzzy sets)**

Adopted from [31], a generalized type-II fuzzy set, $\tilde{A}$, is defined by the following function:

(3) $$\begin{array}{c} \tilde{A}=\int_{x\in X}\frac{\left[\int_{u\in J_{x}}f_{x}\left(u\right)/u\right]}{x}, \end{array}$$

where *J*_*x*_⊆[0,1], *x* ∈ *X*, *u* ∈ [0,1], and *f*_*x*_(*u*)∈[0,1].

The membership grade of a generalized type-II fuzzy set is known as the secondary membership function. This function maps the primary membership grades, $\mu_{\tilde{A}}(x)$, to their respective secondary membership grades.

**Definition 4 (interval type-II fuzzy sets)**

Adopted from [32], an interval type-II fuzzy set, $\tilde{A}$, is defined by following function:

(4) $$\begin{array}{c} \tilde{A}=\int_{x\in X}\frac{\left[\int_{u\in J_{x}}1/u\right]}{x}, \end{array}$$

where *J*_*x*_⊆[0,1], *x* ∈ *X*, and *u* ∈ [0,1].

An interval type-II fuzzy set maps the primary membership function, $\mu_{\tilde{A}}(x)$, to the secondary membership function *f*(*x*). This secondary function maps the value [0,1] to the values in {0,1}. This crisp value of the secondary membership function allows interval type-II fuzzy sets to be processed more efficiently than the generalize type-II fuzzy sets.

## 3. Basic Structure of the Proposed Model

**Figure 1:**
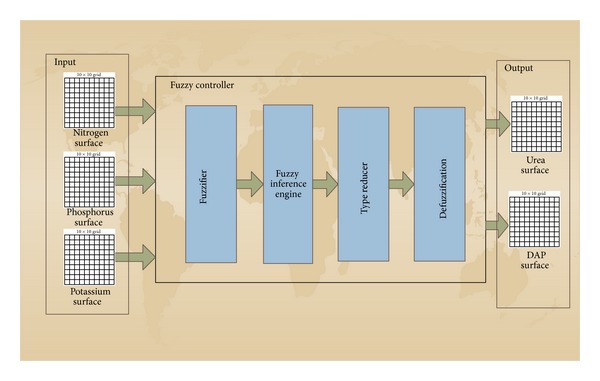
Basic structure of our system.

The system takes the amount of soil nutrients and time of crop production as input. The nutrient includes potassium, phosphorus, and nitrogen. The proposed system fuzzifies these values based on type-II membership functions. The fuzzified values are passed to the fuzzy inference engine where IF-THEN fuzzy rules are applied to get a fuzzy type-II output. This output is passed through the type reducer and reduced to type-I, which is defuzzified to a crisp value. A new surface is generated which provide us the pattern of the use of fertilizer in our area of interest. The algorithm for these steps is given in Algorithm 1 and our system is explained in the diagram in Figure 1.

## 4. Material and Analysis

The dataset consists of four inputs, that is, nitrogen, phosphorous, potassium, and crop time and generates two outputs, that is, urea and DAP. The nitrogen, phosphorous, and potassium are in the form of spatial surfaces that are classified according to the ranges defined by SFRI. These ranges are shown in Tables 1 and 2

These surfaces, along with crop time, are fed into the type-II fuzzy system for processing and interpretating linguistic values and the system results into the development of new surfaces showing the use of fertilizers, that is, urea and DAP.

The type-II fuzzy system uses the Mamdani inference method [9]. It consists of the following units:

- fuzzifier;
- fuzzy inference engine;
- type reducer;
- defuzzifier.

### 4.1. Fuzzifier

**Figure 2:**
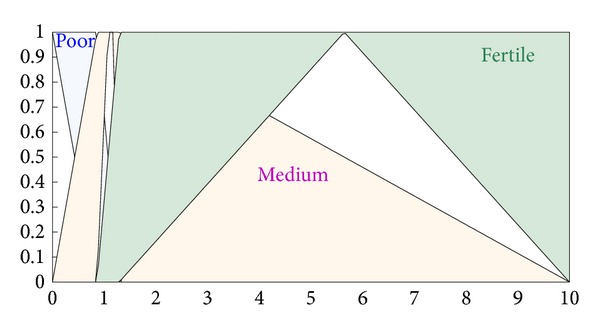
Membership functions for nitrogen.

**Figure 3:**
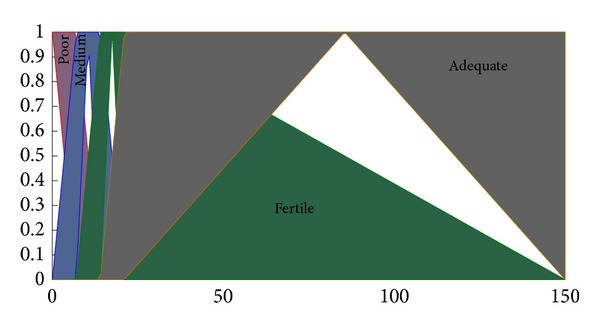
Membership functions for phosphorus.

**Figure 4:**
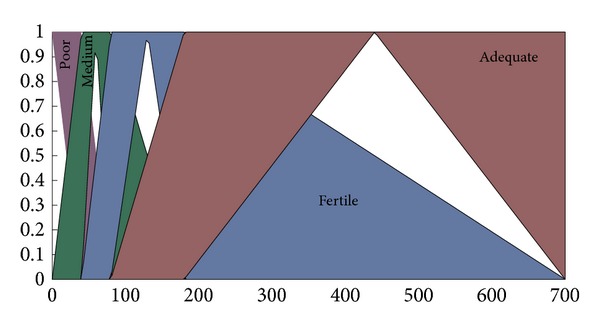
Membership functions for potassium.

**Figure 5:**
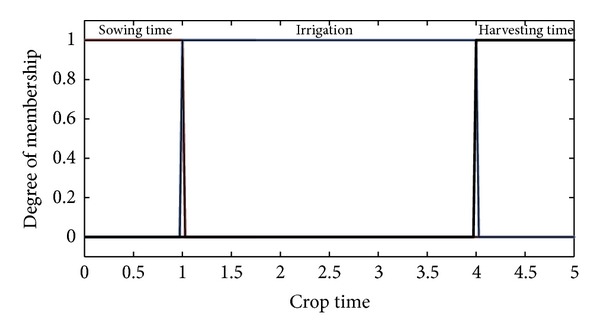
Membership functions for cropping time.

The type-II fuzzifier maps the numeric values of the surface into interval values of the type-II fuzzy set ${\tilde{A}}_{x}$ in *X*, using a membership function defined on the basis of classes defined in Tables 1 and 2. These membership functions are shown in Figures 2, 3, 4, and 5.

The membership functions of the nutrients, that is, nitrogen, phosphorous, and potassium, have footprints of uncertainty (FOU). The FOU of these nutrients is bounded by upper and a lower type-I membership functions denoted by ${\bar{\mu }}_{A}$ and ${\underline{\mu }}_{A}$. The FOU is shown in the shaded area in Figures 2, 3, and 4. This unit fuzzifies the numeric values of the nutrient surfaces and calculates the lower and upper bounds, ${\bar{\mu }}_{A}$ and ${\underline{\mu }}_{A}$, whereas the numeric value of crop time is fuzzified to only a type-I degree of membership as it has no FOU.

### 4.2. Fuzzy Inference Engine

This unit applies the rules on the fuzzy input prepared by fuzzifier. The structure of the rules in the type-II fuzzy inference engine is the same as that of the type-I fuzzy inference engine. The only difference is in the antecedents and consequents of the rules that are represented by the type-II fuzzy sets. These rules are defined in the form of

(5) IF 〈fuzzy  type-II  preposition〉THEN 〈fuzzy  type-II  preposition〉.

The fuzzy type-II preposition defined in the form *x* is *A* where *x* is the linguistic variable and *A* is the linguistic value defined in the form of lower and upper bounds ${\bar{\mu }}_{A}$ and ${\underline{\mu }}_{A}$. In this engine, we have used the rules defined by SFRI Pakistan listed in Table 3.

### 4.3. Type Reducer

This unit takes the output of fuzzy inference engine, which is of in the form of a type-II fuzzy set, and converts it into a type-I fuzzy set. We have used the center of sets type reduction, which produces the output in interval form. The center of type reduction is expressed as

(6) $$\begin{array}{c} Y_{\cos}\left(x\right)=\left[y_{l},y_{r}\right]. \end{array}$$

It is a fact that centroid of interval type-II fuzzy set is an interval type-I fuzzy set. Karnik and Mendel developed a KM algorithm for type reducer which is fast monotonically and exponentially [33].

### 4.4. Defuzzifier

This unit maps the type-I fuzzy set into a crisp number. For defuzzification, we calculate the centroid [34] of the left *y*_*l*_ and right *y*_*r*_ end points of the type reduced set calculated by the KM algorithm, as follows:

(7) $$\begin{array}{c} y\left(x\right)=\frac{y_{l}+y_{r}}{2}. \end{array}$$

**Figure 6:**
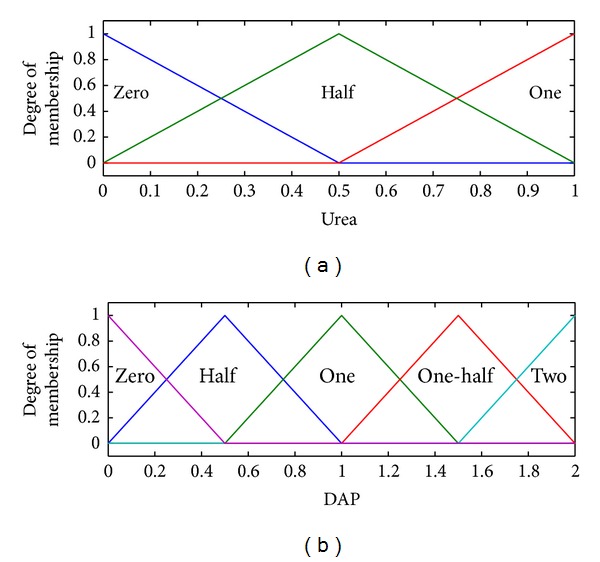
Membership functions for urea and DAP.

The centroid defuzzifies *y*(*x*) into two outputs, that is, urea and diammonium phosphate (DAP). The membership functions for the two outputs are shown in Figure 6.

**Figure 7:**
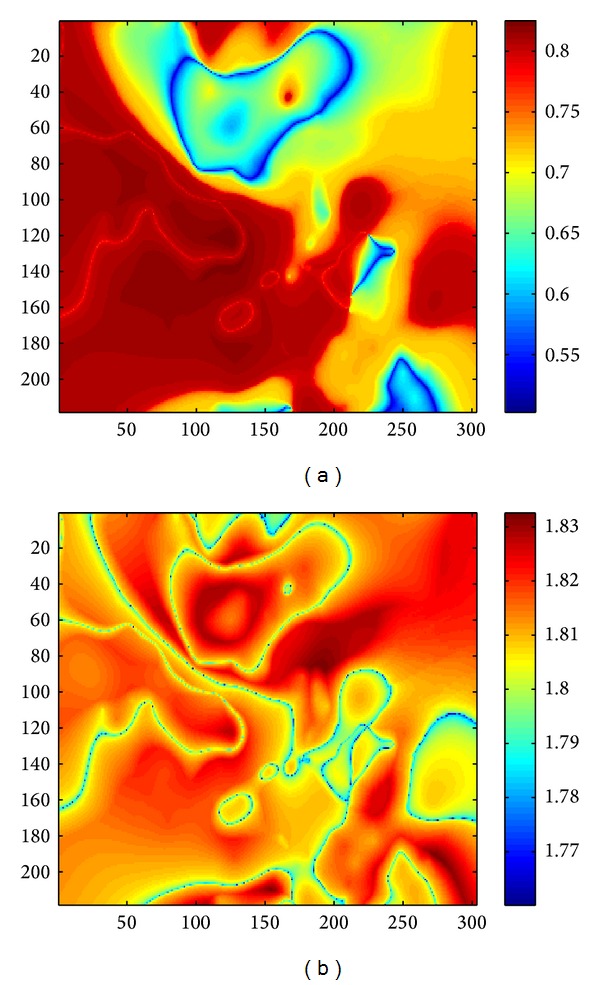
Surfaces of urea and DAP.

The defuzzifier estimates the crisp output value according to the center of gravity method using the mathematical equation ∫*μc* · *z* · *dz*/∫*μc* · *dz*. This output is then assigned to the corresponding pixel of the output surface.  Figure 7 shows the surface of the fertilizer for irrigated wheat at the irrigation time, prepared using type-II algorithm in Section 3.

Figure 7 shows the spatial trend for the use of fertilizers. These images are shown in blue to red ramp. The reddish the area is, the more the fertilizer is needed, and the bluish the area, the less the fertilizer is needed. The range of urea within our area of interest is from 0.5 to 0.85. It means that the red area in the image requires around 0.85 bag of urea and blue area requires 0.5 bag of urea. Similarly, the range of DAP is from 1.76 to 1.84; that is, red area requires 1.76 bags of DAP and blue area requires 1.84 bags of DAP. Please note that these quantities of fertilizers are required at the time of first irrigation, which is the third month after sowing.

## 5. Complexity of Algorithm

**Figure 8:**
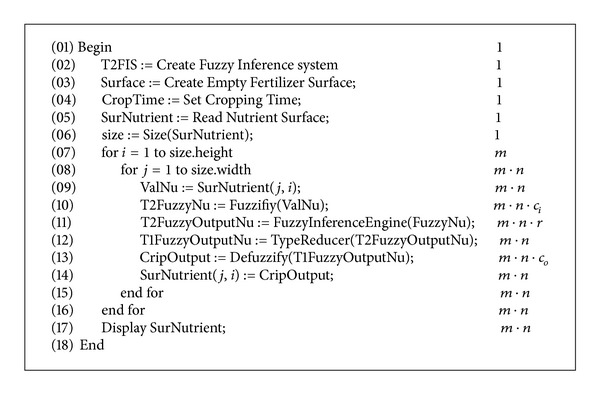
Complexity of algorithm.

The running time of algorithm is *O*(*mnr*), where *m* is the height of the raster, *n* is the width of the raster, and *r* is the number of expert rules. From Figure 8, lines 1–6 are in constant time, as these are declarations of the variables and objects. The running time of lines 7-8 is nested loops of *mn* complexity. Lines 9, 12, and 14 are of constant time. The running time of line 10 depends on the number of input classes *c*_*i*_ (see Tables 1 and 2) used for fuzzification. Similarly, the running time of lines 11 and 13 depends upon the number of rules, *r*, mentioned in Table 3 and the number of output classes *c*_*o*_ shown in Figure 6, respectively. Thus, the running time of the block of lines 9–14 is *O*(*r* + *c*_*i*_ + *c*_*o*_). As this block is nested under loops with growth rate *O*(*mn*), therefore, the running time of the block including loops is *O*(*mn*(*r* + *c*_*i*_ + *c*_*o*_)). Since in our case *c*_*i*_ and *c*_*o*_ are small constants, therefore, the running time of algorithm is *O*(*mnr*).

## 6. Conclusions

Most of the spatial entities and phenomena are defined in linguistic terms. These uncertain behaviors of spatial entities can only be defined by fuzzy sets and their generalizations. A type-II fuzzy set is an extension of the fuzzy set that represents uncertainty by an additional dimension. This extra dimension gives more freedom for representing uncertainty. In this paper, we have shown the use of type-II fuzzy sets for developing a fuzzy system for the generation of a fertilizer surface within a specified spatial extent, given nutrients of soil and cropping time. The type-II fuzzy system provides the capability of handling a higher level of uncertainty in defining the linguistic classes of nutrients in soil. Though the analysis presented in this paper is only for irrigated wheat crop, they clearly depict that the use of a type-II fuzzy inference system in a GIS can help identify the spatial patterns of the requirement of fertilizers for any crop. This paper has presented a basic platform for the development of spatial surfaces using a type-II fuzzy inference engine based on human linguistic values. The running time of Algorithm 1 is *O*(*mnr*), where *m* is the height of the raster, *n* is the width of the raster, and *r* is the number of expert rules. 95 rules were used in this fuzzy inference system, resulting in slow execution time. Research can be conducted on reducing the rules using different available methods like SVD, combo, or singleton. We have also used Mamdani type fuzzy system which can also be tested and compared with Sugeno type fuzzy system.

## Figures and Tables

**Algorithm 1:**
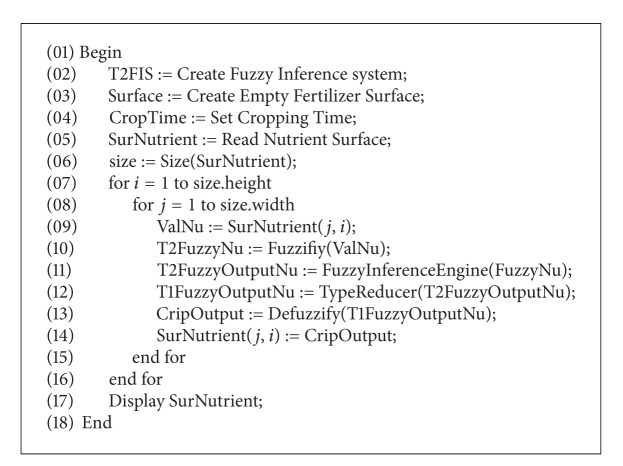
Algorithm for fuzzy decision support system for fertilizer.

**Table:** (a)

	Poor (P)	Medium (M)	Fertile (F)
Nitrogen	0 to 0.86	0.86 to 1.29	1.29 to 10

**Table:** (b)

	Poor (P)	Medium (M)	Fertile (F)	Adequate (A)
Phosphorus	0 to 7	7 to 14	14 to 21	21 to 150

**Table:** (c)

	Poor (P)	Medium (M)	Fertile (F)	Adequate (A)
Potassium	0 to 40	40 to 80	80 to 180	180 to 700

**Table 2:** Ranges and linguistic value of the input variable cropping time.

	Sowing	Irrigation	Harvesting
Crop time	November to December	Late December to late February	March to April

**Table 3:** Fuzzy rules for irrigated wheat.

Input				Output
Nitrogen	Phosphorous	Potassium	Cropping time	Fertilizer
Poor	Poor	Poor	Sowing	0.5 bag of urea, 2 bags of DAP
Poor	Poor	Poor	Irrigation	1 bag of urea
Poor	Poor	Medium	Sowing	0.5 bag of urea, 2 bags of DAP
Poor	Poor	Fertile	Sowing	0.5 bag of urea, 2 bags of DAP
Poor	Poor	Fertile	Irrigation	1 bag of urea
Poor	Poor	Adequate	Sowing	0.5 bag of urea, 2 bags of DAP
Poor	Poor	Adequate	Irrigation	1 bag of urea
Poor	Medium	Poor	Sowing	0.5 bag of urea, 1.5 bags of DAP
Poor	Medium	Poor	Irrigation	1 bag urea
Poor	Medium	Medium	Sowing	0.5 bag of urea, 1.5 bags of DAP
Poor	Medium	Medium	Irrigation	1 bag of urea
Poor	Medium	Fertile	Sowing	0.5 bag of urea, 1.5 bags of DAP
Poor	Medium	Fertile	Irrigation	1 bag of urea
Poor	Medium	Adequate	Sowing	0.5 bag of urea, 1.5 bags of DAP
Poor	Medium	Adequate	Irrigation	1 bag of urea
Poor	Fertile	Poor	Sowing	0.5 bag of urea, 1 bag of DAP
Poor	Fertile	Poor	Irrigation	1 bag of urea
Poor	Fertile	Medium	Sowing	0.5 bag of urea, 1 bag of DAP
Poor	Fertile	Medium	Irrigation	1 bag of urea
Poor	Fertile	Fertile	Sowing	0.5 bag of urea, 1 bag of DAP
Poor	Fertile	Fertile	Irrigation	1 bag of urea
Poor	Fertile	Adequate	Sowing	0.5 bag of urea, 1 bag of DAP
Poor	Fertile	Adequate	Irrigation	1 bag of urea
Poor	Adequate	Poor	Sowing	0.5 bag of urea
Poor	Adequate	Poor	Irrigation	1 bag of urea
Poor	Adequate	Medium	Sowing	0.5 bag of urea
Poor	Adequate	Medium	Irrigation	1 bag of urea
Poor	Adequate	Fertile	Sowing	0.5 bag of urea
Poor	Adequate	Fertile	Irrigation	1 bag of urea
Poor	Adequate	Adequate	Sowing	0.5 bag of urea
Poor	Adequate	Adequate	Irrigation	1 bag of urea
Medium	Poor	Poor	Sowing	0.5 bag of urea, 2 bags of DAP
Medium	Poor	Poor	Irrigation	1 bag of urea
Medium	Poor	Medium	Sowing	0.5 bag of urea, 2 bags of DAP
Medium	Poor	Medium	Irrigation	1 bag of urea
Medium	Poor	Fertile	Sowing	0.5 bag of urea, 2 bags of DAP
Medium	Poor	Fertile	Irrigation	1 bag of urea
Medium	Poor	Adequate	Sowing	0.5 bag of urea, 2 bags of DAP
Medium	Poor	Adequate	Irrigation	1 bag of urea
Medium	Medium	Poor	Sowing	0.5 bag of urea, 1.5 bags of DAP
Medium	Medium	Poor	Irrigation	1 bag of urea
Medium	Medium	Medium	Sowing	0.5 bag of urea, 1.5 bags of DAP
Medium	Medium	Medium	Irrigation	1 bag of urea
Medium	Medium	Fertile	Sowing	0.5 bag of urea, 1.5 bags of DAP
Medium	Medium	Fertile	Irrigation	1 bag of urea
Medium	Medium	Adequate	Sowing	0.5 bag of urea, 1.5 bags of DAP
Medium	Medium	Adequate	Irrigation	1 bag of urea
Medium	Fertile	Poor	Sowing	0.5 bag of urea, 1 bag of DAP
Medium	Fertile	Poor	Irrigation	1 bag of urea
Medium	Fertile	Medium	Sowing	0.5 bag of urea, 1 bag of DAP
Medium	Fertile	Medium	Irrigation	1 bag of urea
Medium	Fertile	Fertile	Sowing	0.5 bag of urea, 1 bag of DAP
Medium	Fertile	Fertile	Irrigation	1 bag of urea
Medium	Fertile	Adequate	Sowing	0.5 bag of urea, 1 bag of DAP
Medium	Fertile	Adequate	Irrigation	1 bag of urea
Medium	Adequate	Poor	Sowing	0.5 bag of urea
Medium	Adequate	Poor	Irrigation	1 bag of urea
Medium	Adequate	Medium	Sowing	0.5 bag of urea
Medium	Adequate	Medium	Irrigation	1 bag of urea
Medium	Adequate	Fertile	Sowing	0.5 bag of urea
Medium	Adequate	Fertile	Irrigation	1 bag of urea
Medium	Adequate	Adequate	Sowing	0.5 bag of urea
Medium	Adequate	Adequate	Irrigation	1 bag of urea
Fertile	Poor	Poor	Sowing	0.5 bag of urea, 2 bags of DAP
Fertile	Poor	Poor	Irrigation	0.5 bag of urea
Fertile	Poor	Medium	Sowing	0.5 bag of urea, 2 bags of DAP
Fertile	Poor	Medium	Irrigation	0.5 bag of urea
Fertile	Poor	Fertile	Sowing	0.5 bag of urea, 2 bags of DAP
Fertile	Poor	Fertile	Irrigation	0.5 bag of urea
Fertile	Poor	Adequate	Sowing	0.5 bag of urea, 2 bags of DAP
Fertile	Poor	Adequate	Irrigation	0.5 bag of urea
Fertile	Medium	Poor	Sowing	0.5 bag of urea, 1.5 bags of DAP
Fertile	Medium	Poor	Irrigation	0.5 bag of urea
Fertile	Medium	Medium	Sowing	0.5 bag of urea, 1.5 bags of DAP
Fertile	Medium	Medium	Irrigation	0.5 bag of urea
Fertile	Medium	Fertile	Sowing	0.5 bag of urea, 1.5 bags of DAP
Fertile	Medium	Fertile	Irrigation	0.5 bag of urea
Fertile	Medium	Adequate	Sowing	0.5 bag of urea, 1.5 bags of DAP
Fertile	Medium	Adequate	Irrigation	0.5 bag of urea
Fertile	Fertile	Poor	Sowing	0.5 bag of urea, 1 bag of DAP
Fertile	Fertile	Poor	Irrigation	0.5 bag of urea
Fertile	Fertile	Medium	Sowing	0.5 bag of urea, 1 bag of DAP
Fertile	Fertile	Medium	Irrigation	0.5 bag of urea
Fertile	Fertile	Fertile	Sowing	0.5 bag of urea, 1 bag of DAP
Fertile	Fertile	Fertile	Irrigation	0.5 bag of urea
Fertile	Fertile	Adequate	Sowing	0.5 bag of urea, 1 bag of DAP
Fertile	Fertile	Adequate	Irrigation	0.5 bag of urea
Fertile	Adequate	Poor	Sowing	0.5 bag of urea
Fertile	Adequate	Poor	Irrigation	0.5 bag of urea
Fertile	Adequate	Medium	Sowing	0.5 bag of urea
Fertile	Adequate	Medium	Irrigation	0.5 bag of urea
Fertile	Adequate	Fertile	Sowing	0.5 bag of urea
Fertile	Adequate	Fertile	Irrigation	0.5 bag of urea
Fertile	Adequate	Adequate	Sowing	0.5 bag of urea
Fertile	Adequate	Adequate	Irrigation	0.5 bag of urea
